# Hepatoprotective Activity of Vitamin E and Metallothionein in Cadmium-Induced Liver Injury in *Ctenopharyngodon idellus*

**DOI:** 10.1155/2018/9506543

**Published:** 2018-04-11

**Authors:** Yajiao Duan, Jing Duan, Yang Feng, Xiaoli Huang, Wei Fan, Kaiyu Wang, Ping Ouyang, Yongqiang Deng, Zongjun Du, Defang Chen, Yi Geng, Shiyong Yang

**Affiliations:** ^1^Department of Aquaculture, Sichuan Agricultural University, Wenjiang, Sichuan 611130, China; ^2^Neijiang City Academy of Agricultural Sciences, Neijiang, Sichuan 641000, China; ^3^College of Animal Science and Veterinary Medicine, Sichuan Agricultural University, Wenjiang, Sichuan 611130, China; ^4^Animal Disease Prevention and Control Center of Sichuan Province, Chengdu, Sichuan 610000, China

## Abstract

As an environmental and industrial pollutant, cadmium (Cd) can cause a broad spectrum of toxicological effects. Multiple organs, especially the liver, are considerably affected by Cd in both humans and animals. We investigated the protective effects of metallothionein (MT) and vitamin E (VE) supplementation on Cd-induced apoptosis in the grass carp (*Ctenopharyngodon idellus*) liver. Grass carp were divided into four groups: the control group, Cd + phosphate-buffered saline (PBS) group, Cd + VE group, and Cd + MT group. All fish were injected with CdCl_2_ on the first day and then VE, MT, and PBS were given 4 days postinjection, respectively. The results showed that Cd administration resulted in liver poisoning in grass carp, which was expressed as an increase in Cd contents, malondialdehyde (MDA) concentration, percentage of hepatocyte apoptosis, and apoptosis-related gene mRNA transcript expression. However, VE and MT treatments protected against Cd-induced hepatotoxicity in grass carp by decreasing Cd contents, lipid peroxidation, and histological damage and reducing the percentage of hepatocyte apoptosis by regulating related mRNA transcript expression. These data demonstrate that oxidative stress and activation of the caspase signaling cascade play a critical role in Cd-induced hepatotoxicity. However, VE and MT alleviate Cd-induced hepatotoxicity through their antioxidative and antiapoptotic effects, and MT has a more powerful effect than VE.

## 1. Introduction

Cadmium (Cd) is a widespread environmental toxin and occupational pollutant and was listed as one of the most toxic substances to human health and the most malicious carcinogen by the Agency for Toxic Substances and Disease Registry and the International Agency for Research on Cancer, respectively [1, 2]. Cd can endanger human and animal health by causing multiple organ damage [3–5], which may result in prostate, lung, and testes cancer in humans [6] and kidney, liver, bone, and brain injury, as well as immune and cardiovascular system impairment [7].

Free Cd, which can cause human poisoning, is mainly taken into the body via food [8]. Fish is known to be the richest source of toxic trace elements in humans, and these animals live in contaminated water and bioaccumulate Cd through the food chain [9]. Cd generally enters an aquatic organism's body, accumulates in the body via the blood circulation, and binds to the sulfhydryl groups of proteins [10]. Thus, Cd can be transferred to humans following the ingestion of aquatic organisms. This issue is critical in Cd intoxication research, not only for the aquaculture industry but also for human health [9].

The mechanisms of Cd-induced damage in organisms are mainly related to apoptosis and oxidative stress [11–16]. Glutathione [17], cysteine [10], anthocyanin [18], zinc and selenium [19], and other drugs have been found to protect organisms against Cd-induced damage by reducing apoptosis and blocking oxidative stress pathways. Vitamin E (VE) is a primary liposoluble antioxidant, which may play an important role in scavenging free oxygen radicals and stabilizing cell membranes, thus maintaining cell permeability [20]. In addition, metallothionein (MT), a ubiquitous metal-binding protein, is an endogenous detoxification factor in organisms and is highly conserved in evolution, and can reduce damage by chelating Cd [21]. Furthermore, the translocation of MT to the nucleus is probably associated with the protection of cells by resisting DNA damage and apoptosis as well as gene transcription during different stages of the cell cycle [22]. However, it is unclear whether MT can protect fish from damage through these pathways. It is necessary to determine whether VE and MT have protective effects and which drug has the best effect against Cd-induced damage in fish.

Liver is one of the primary target organs of Cd and is extremely sensitive to both acute and chronic Cd exposures [23]. Grass carp (*Ctenopharyngodon idellus*) aquaculture accounts for 18.10% of the total products from freshwater fisheries worldwide each year [24]. Cd-induced chronic diseases such as rickets, deformities, and liver toxicity can seriously jeopardize the grass carp cultivation industry [25]. Therefore, we have created an “intoxication–detoxification” comparative grass carp model to determine whether Cd-induced liver injury can be prevented by exogenous VE and MT supplementation. The underlying protective mechanism was also investigated.

## 2. Materials and Methods

### 2.1. Chemicals and Their Preparation

CdCl_2_ was obtained from MOLBASE (Shanghai) Biotechnology Co. Ltd. VE (liposoluble; purity 99%) and MT-2 (pure rabbit liver MT powder; purity 99%) were purchased from Shanghai Shou Feng Industrial Co. Ltd. and Sigma-Aldrich Company (Beijing, China), respectively. All other chemicals were of analytical grade or the highest grade available and obtained from local companies.

CdCl_2_ and MT were dissolved in sterilized PBS. Liposoluble VE was dissolved in sterilized phosphate-buffered saline (PBS) and emulsified using an ultrasonic crusher until milky.

### 2.2. Experimental Carp

Grass carp of similar weight (50 ± 3.4 g) and length (15 ± 2.5 cm) were purchased from a fish farm in Meishan, Sichuan, China. The fish were acclimatized in the laboratory for 2 weeks before experimentation. The carp were exposed to a light : dark cycle of 12 h : 12 h, an uninterrupted oxygen supply to ensure more than 5 mg/L dissolved oxygen, pH of 6.5–8.5, ammoniacal nitrogen and nitrite maintained at 0–0.02 mg/L, the water in the tanks was pretreated with UV light and an aeration process, and 20% of the culture water was renewed every day. The fish were fed with commercial pellets (Tongyi Company, Suzhou, China) twice a day for 2 weeks. Fish that has a bright body color and is responsive, robust, and healthy were selected for experimentation. All animal handling procedures were approved by the Animal Care and Use Committee of Sichuan Agricultural University, following the guidelines of the animal experiments of Sichuan Agricultural University, under permit number DY-S20144657.

### 2.3. Establishment of the “Intoxication–Detoxification” Model

#### 2.3.1. Determination of the LC_50_ of Cd in Grass Carp

To assess the lethal concentration 50% (LC_50_) of CdCl_2_, similar body weight (54 ± 4.2 g) grass carp (*n* = 60) were divided into six groups (10 fish/group). Our previous study found that large amounts of precipitate covered the surface of skin and gills and led to dyspnea during CdCl_2_ contaminated water; therefore, CdCl_2_ was administered by intraperitoneal injection. The nominal concentrations of Cd tested were 71.308, 101.696, 145.036, 206.845, 294.994, and 420.709 *μ*mol/kg. The experiment in each group was repeated three times, and the number of dead fish in 96 h was recorded. No food was provided during the test. The LC_50_ was determined with the Karber method [26]. The results showed that the mortality rate rose as the concentration increased within 96 h in the different groups. No deaths were recorded in the control group. According to the modified Kobvgguffer method [27], the LC_50_ was 199.631 *μ*mol/kg (Table 1).

#### 2.3.2. Challenge and Detoxification Reagent Injection

**Figure 1:**
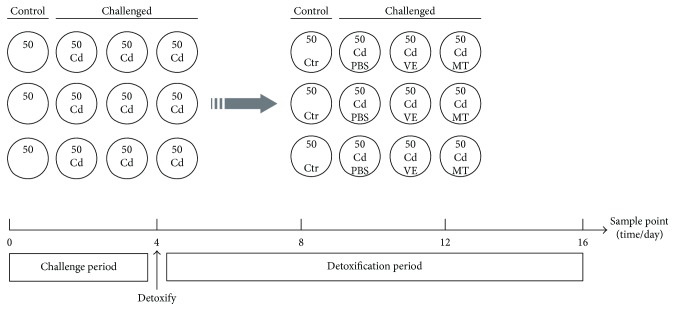
Study design showing the challenge model based on a three-parallel-tank system. Each group included 150 fish divided into three parallel tanks.

As previously reported [28], 1/10 LC_50_ was used as the subacute concentration in the present study. Challenged fish (*n* = 450) were divided into three groups and each group included three parallel tanks. Another 150 control fish were also divided into three parallel tanks (Figure 1). CdCl_2_ was injected into the challenged fish (*n* = 450) intraperitoneally during the challenge period. Healthy fish (*n* = 150) not given CdCl_2_ were included as controls. On the 4th day post Cd injection, challenged fish were medicated with 4 mL/kg PBS, 20 IU/kg VE [29], and 2.1 mg/kg MT [30], respectively. Liver samples were collected after treatment on the 4th, 8th, 12th, and 16th days postchallenge.

### 2.4. Histological Analysis

Six fish in each group were necropsied at 4, 8, 12, and 16 days. Liver tissues were fixed in 10% neutral formalin and routinely processed in paraffin. Liver tissues were also trimmed into cassettes, dehydrated in graded ethanol solutions, cleared in xylene, and embedded in paraffin wax. Sections of 5 *μ*m for hematoxylin and eosin (H&E) staining were prepared prior to microscopic analysis.

### 2.5. Determination of the Cd Content in the Liver

All glass containers used in the trial were soaked in 10% nitric acid for 24 h and then rinsed several times with deionized water. The grass carp liver (*n* = 6) was sampled on the 4th, 8th, 12th, and 16th days, treated immediately with liquid nitrogen, and then stored at −80°C. The liver tissue was placed in a 25 mL stopper flask, and a few clean glass spheres were added to prevent splashing. The tissue was incubated with 2 mL mixed acid (HNO_3_ : HClO_4_ = 4 : 1) overnight and then transferred to an 18°C sand bath to digest, until the color of the liquid was clear. The liver tissue was then transferred to a volumetric flask and detected using an AA680 Shimadzu (Kyoto, Japan) flame atomic absorption spectrometer [31, 32], under the following conditions: wavelength of 228.8 nm, crack 0.5 nm, lamp current 6 mA, drying at 100°C for 20 s, ashing at 300°C for 15–20 s and an atomization at 1500°C. The results were calculated according to the following formula:

(1) $$\begin{array}{c} X=\frac{\left(A1-A2\right)\times V\times 1000}{M\times 1000}, \end{array}$$

where *X* (*μ*g/kg) is the Cd content in the liver, A1 (*μ*g/mL) is the Cd content in the sample, A2 (*μ*g/mL) is the Cd content in the reagent blank, *V* (mL) is the total volume after sample treatment, and *M* (g) is the sample mass.

### 2.6. Determination of the MDA Content in the Liver

MDA is a breakdown product of the oxidative degradation of cell membrane lipids and is generally considered an indicator of lipid peroxidation [33]. Lipid peroxidation was evaluated by measuring MDA concentrations using a spectrophotometer to determine the color produced during the reaction of thiobarbituric acid with MDA. At 4, 8, 12, and 16 days, six fish in each group were anesthetized and immediately necropsied. Livers were immediately removed and stored at 0°C in 0.65% NaCl solution. Approximately 1 g liver tissue was homogenized with 9 mL 0.65% NaCl solution in a homogenizer on ice. The homogenates were then centrifuged at 3500*g* at 4°C, and total protein in the supernatant was determined with a protein quantification kit (A045-2) (NJJCBIO, Nanjing, China). The activity of MDA (A003-2) in the supernatant was determined using a commercial kit (NJJCBIO, Nanjing, China), according to the manufacturer's instructions.

### 2.7. Liver Cell Apoptosis Measurement

At 4, 8, 12, and 16 days, six fish from each group were anesthetized by MS-222. The livers were then sampled to determine the percentage of apoptotic cells using flow cytometry [34]. The livers were immediately minced to form a cell suspension and filtered through a 300-mesh nylon screen. Cells were washed twice with cold PBS, and the cell pellet was resuspended at a concentration of 1 × 10^6^ cells/mL in PBS. Then, 5 *μ*L of annexin V-fluorescein isothiocyanate (V-FITC) (BD Pharmingen, Franklin Lakes, New Jersey, USA) and 5 *μ*L of propidium iodide (PI) (BD Pharmingen) were added into the 100 *μ*L cell suspension, respectively. The cells were then incubated with annexin V-FITC/PI in the dark for 15 min at room temperature. Apoptotic cells were examined by flow cytometry (BD FACSCalibur). The data were analyzed using Expo 32 software (Beckman Coulter, Kraemer Boulevard, Brea, California, USA).

### 2.8. Quantitative Real-Time Polymerase Chain Reaction (qPCR) Analysis

At 4, 8, 12, and 16 days, six fish from each group were anesthetized by MS-222. Livers were sampled, placed in RNA/DNA protector solution (TaKaRa, Dalian, China), and stored at 4°C. The livers were then homogenized by crushing with a mortar and pestle and stored at −80°C.

Total RNA was isolated from livers with a TRIzol reagent (TaKaRa). Complementary DNA (cDNA) was synthetized from 1 *μ*g of RNA using the PrimeScript™ RT reagent kit with gDNA eraser (RR047A, TaKaRa). qPCR was performed using an SYBR green real-time PCR kit (TaKaRa, Kusatsu, Japan) and a thermocycler (Bio-Rad, Hercules, California, USA). *β-Actin* and *18S ribosomal RNA* were used as reference genes to determine the relative expression of target genes, which was the invariant expression in the grass carp liver in our previous validation. The primers used for qPCR are listed in Table 2.

For qPCR, the 25 *μ*L reaction mixture contained 12.5 *μ*L SYBR green PCR master mix, 8.5 *μ*L diethylpyrocarbonate-treated water, 1.0 *μ*L of forward primer, 1.0 *μ*L of reverse primer, and 2 *μ*L cDNA. The following program conditions were used for the reactions: 3 min at 95°C for 1 cycle, samples were amplified for 40 cycles at 95°C for 10 s, melting temperature of a specific primer pair for 30 s, followed by 10 s at 95°C, and 72°C for 10 s. To distinguish between specific and nonspecific reaction products, a melting curve was obtained at the end of each run. The 2^−ΔΔCT^ method was used to calculate relative changes in mRNA transcript expression from the qPCR results (ΔCT = CT_target gene_ − CT_*β*−actin_, ΔΔCT = ΔCT_experimental_ − ΔCT_control_) [35].

### 2.9. Statistical Analysis

The results are expressed as the mean value (*n* = 6) and standard deviation. The significance of differences was analyzed by variance analysis. The analysis was performed using one-way analysis of variance while the *t*-test was applied to determine whether the differences between groups were significant (SPSS v.20.0, IBM Corp., Armonk, New York, USA). A value of *P* < 0.05 was considered significant, while a *P* < 0.01 was considered highly significant.

## 3. Results

### 3.1. VE and MT Protected Cell Morphological Integrity in the Liver

**Figure 2:**
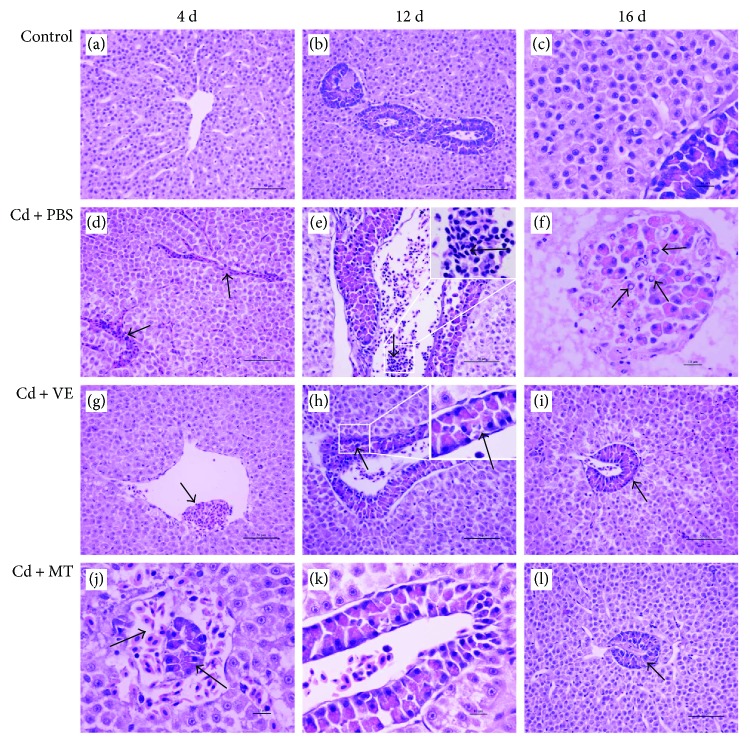
Histopathological changes in the livers induced by cadmium and detoxification-treated livers collected on the 4th, 12th, and 16th days after challenge with cadmium and treatment with VE and MT. (a–c) The liver of the control group. (d–f) Histopathological changes in the liver in the PBS group with time. (d) The arrow shows congestion in hepatic sinusoids. (e) The number of inflammatory cells increased in the blood vessels and hepatic sinusoids on the 12th and 16th days postchallenge. The arrow shows inflammatory cells. (f) Necrotic and apoptotic cells present in pancreatic cells. The arrows show cell degeneration and necrosis. (g–i) Histopathological changes in the liver in the VE group with time. (g) Hyperemia did not significantly improve during the Cd challenge in the VE group on the 4th day. The arrows show red blood cells. (h, i) Hepatopancreas recovered on the 12th and 16th days in the VE group. The arrows show pancreatic cells restored. (j–l) Histopathological changes in the liver in the MT group with time. (j) Hyperemia did not significantly improve during the Cd challenge in the MT group on the 4th day. The arrows show pancreas congestion. (k, l) Inflammatory cells decreased on the 12th and 16th days and hepatopancreas damage recovered. The arrows show red cells in the pancreas decreased.

Under normal conditions, grass carp hepatocytes had normal morphology and growth, and there were no significant morphological changes in the hepatopancreas (Figures 2(a)–2(c)). After Cd treatment, hepatic sinusoids were congested on the 4th day (Figure 2(d)). On the 12th day, the number of inflammatory cells was elevated in the blood vessels and hepatic sinusoids (Figure 2(e)). Interstitial edema was also noted following Cd challenge. In the final stage of the challenge, various degrees of necrosis and apoptosis in hepatic cells and pancreatic cells were observed (Figure 2(f)).

No obvious congestion in hepatic sinusoids was seen in the VE and MT groups (Figures 2(g) and 2(j)). Inflammatory cells gradually decreased with prolonged detoxification (Figures 2(h) and 2(k)). Simultaneously, interstitial edema in hepatopancreatic tissues also recovered following 12 days of detoxification. In addition, apoptotic and necrotic hepatic cells and pancreatic cells were significantly reduced and gradually recovered (Figures 2(i) and 2(l)).

### 3.2. MT, but Not VE, Decreased Cd Accumulation to Protect the Liver

**Figure 3:**
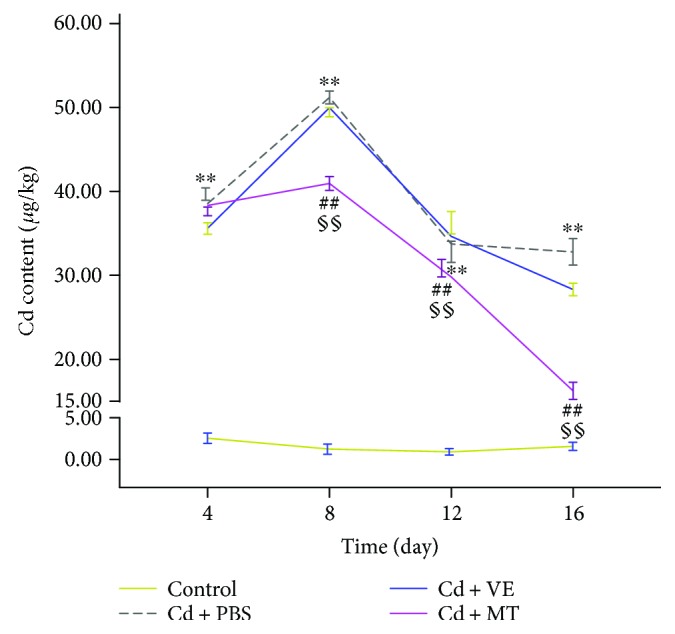
Change in the Cd content (*μ*g/kg wet weight) in the liver of grass carp in four experimental groups. Data are presented as means ± standard deviation. ^∗^*P* < 0.05 or ^∗∗^*P* < 0.01 represents a significant difference or highly significant difference between the control group and the PBS group. ^#^*P* < 0.05 or ^##^*P* < 0.01 represents a significant difference or highly significant difference between the PBS group and the VE/MT group. ^§^*P* < 0.05 or ^§§^*P* < 0.01 represents a significant difference or a highly significant difference between the VE group and the MT group. *n* = 6 in each group.

To further assess the protective effect of VE and MT in the liver, we evaluated the Cd content in the liver under different treatments. Significant accumulation of Cd was seen in the liver after the Cd challenge (*P* < 0.01) compared with the control group, which reached a maximum value on the 8th day (Figure 3). After detoxification, the VE group showed no significant difference in the Cd content compared with the PBS group, which indicated that VE may protect the liver by a different mechanism, such as antioxidation. However, Cd accumulation in the liver in the MT group was significantly reduced compared with that in the PBS group (*P* < 0.01). Furthermore, the Cd content in the VE group was not statistically different to that in the MT group until the 8th day.

### 3.3. MT Is Better Than VE in Protecting the Liver by Eliminating Cd-Induced Lipid Peroxidation

**Figure 4:**
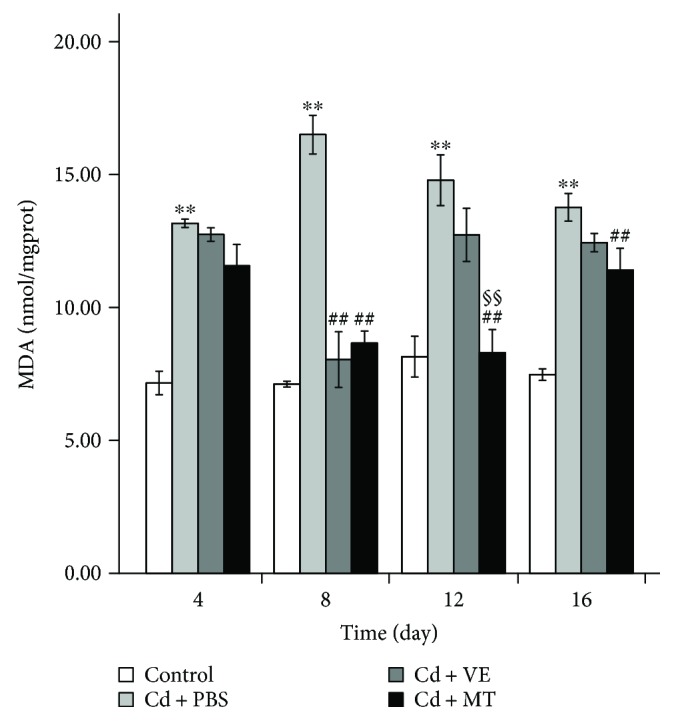
Assessment of MDA in the liver of grass carp in different groups. Data are presented as means ± standard deviation. ^∗^*P* < 0.05 or ^∗∗^*P* < 0.01 represents a significant difference or highly significant difference between the control group and the PBS group. ^#^*P* < 0.05 or ^##^*P* < 0.01 represents a significant difference or highly significant difference between the PBS group and the VE/MT group. ^§^*P* < 0.05 or ^§§^*P* < 0.01 represents a significant difference or a highly significant difference between the VE group and the MT group. *n* = 6 in each group.

Following the Cd challenge, there was significant accumulation of MDA in the liver of grass carp injected with CdCl_2_ (Figure 4). As the Cd challenge time increased, the MDA content in the PBS group increased up to the 8th day and then declined, which indicated that the liver was also involved in detoxification, although the effect was minimal. During detoxification, it was apparent that VE and MT hastened the recovery of MDA; however, MT was superior to VE in this recovery. Both VE and MT significantly reduced the MDA content compared with PBS (*P* < 0.01) on the 8th day. Although this ability declined in the late stages of detoxification, the effect of MT was also significant compared with that of PBS. However, the effect of VE on reducing MDA was only statistically significant after the 12th day (Figure 4). Furthermore, MDA in the MT group was significantly different to that in the VE group after the 12th day.

### 3.4. MT Had a Greater Effect Than VE in Inhibiting Cd-Induced Apoptosis of Liver Cells in Hepatoprotection

**Figure 5:**
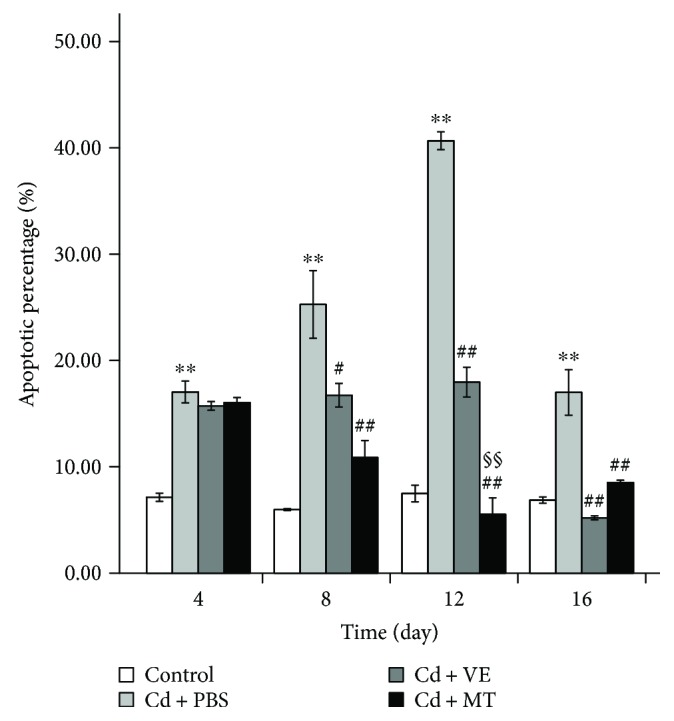
Percentage changes in liver cell apoptosis. Data are presented as means ± standard deviation. ^∗^*P* < 0.05 or ^∗∗^*P* < 0.01 represents a significant difference or highly significant difference between the control group and the PBS group. ^#^*P* < 0.05 or ^##^*P* < 0.01 represents a significant difference or highly significant difference between the PBS group and the VE/MT group. ^§^*P* < 0.05 or ^§§^*P* < 0.01 represents a significant difference or a highly significant difference between the VE group and the MT group. *n* = 6 in each group.

Apoptosis was detected by flow cytometry. Liver cells were examined by counting the total percentage of early apoptotic liver cells and late apoptotic liver cells (Figure 5). Cd induced apoptosis during the challenge period in the PBS group compared with the controls (*P* < 0.01). However, apoptosis was reduced following detoxification by VE and MT in the Cd-challenged group (*P* < 0.01), which indicated that VE and MT played a role in the inhibition of liver cell apoptosis caused by Cd. The PBS group also showed recovery of liver cell apoptosis at the end of the Cd challenge, and both VE and MT hastened this recovery on the 8th day.

### 3.5. VE and MT Reversed the Expression of Apoptosis-Related Genes to Inhibit Apoptosis

**Figure 6:**
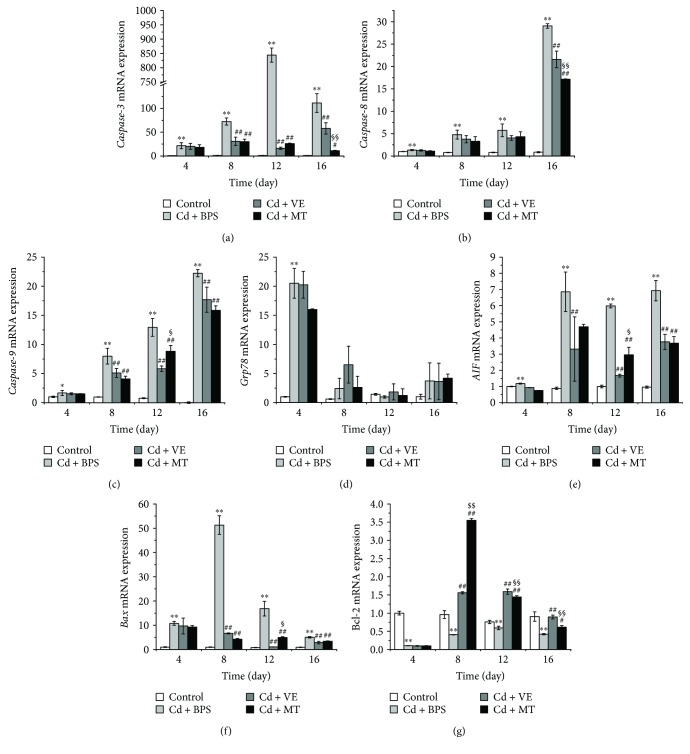
The mRNA expression levels of *caspase-3*, *caspase-8*, *caspase-9*, *Grp78*, *AIF*, *Bax*, and *Bcl-2* in the liver. (a) *Caspase-3*, (b) *caspase-8*, (c) *caspase-9*, (d) *Grp78*, (e) *AIF*, (f) *Bax*, and (g) *Bcl-2*. Data are presented as means ± standard deviation. ^∗^*P* < 0.05 or ^∗∗^*P* < 0.01 represents a significant difference or highly significant difference between the control group and the PBS group. ^#^*P* < 0.05 or ^##^*P* < 0.01 represents a significant difference or highly significant difference between the PBS group and the VE/MT group. ^§^*P* < 0.05 or ^§§^*P* < 0.01 represents a significant difference or a highly significant difference between the VE group and the MT group. *n* = 6 in each group.

To investigate the mechanism of MT and VE in inhibiting hepatocyte apoptosis to protect the grass carp liver, we determined the mRNA transcript expression of several apoptosis-related genes at different times. Compared with the control group, *caspase-3* mRNA expression in the PBS group was significantly increased up to the 12th day and then sharply decreased in the last few days of the Cd challenge. Compared with the PBS group, *caspase-3* expression was downregulated in the VE and MT groups on the 12th and 16th days (Figure 6(a)), and there was a significant difference between the VE/MT group and the PBS group on the 12th day (*P* < 0.01). These results suggest that VE and MT play a role in inhibiting apoptosis as they both inhibited *caspase-3* expression, which is a widely accepted apoptotic terminal gene [36]. Therefore, the mRNA transcript expression of *caspase-3* in the MT group was significantly different to that in the VE group on the 16th day.

To better understand how VE and MT regulate *caspase-3* expression, we detected several main genes of three major apoptotic pathways [37]. These three genes, *caspase-8*, *caspase-9*, and *Grp78/BiP*, showed a high level of mRNA transcript expression after the Cd challenge (Figures 6(b)–6(d). However, *caspase-8* showed no significant change until the 16th day, and *Grp78/BiP* mRNA expression only increased early in the Cd challenge. This may indicate that Cd-induced apoptosis was mainly through the mitochondrial pathway rather than the death receptor pathway and endoplasmic reticulum stress pathway. VE and MT reduced the expression of *caspase-9* but not *caspase-8* or *Grp78*/*BiP* to diminish the expression of *caspase-3* (*P* < 0.01).

We also investigated other apoptosis-related genes during the Cd challenge and VE/MT stimulation to confirm our findings that VE/MT inhibit liver cell apoptosis. Compared with the controls, the mRNA transcript expression of *AIF* (Figure 6(e)) and *Bax* (Figure 6(f)) was significantly enhanced in the PBS group after the Cd challenge, despite the fact that the expression of *AIF* increased continuously, while *Bax* increased up to the 8th day and then declined. Compared with the PBS group, a significant decrease (*P* < 0.01) in *Bax* expression in the VE and MT groups was observed in the liver of grass carp from the 8th to the 16th day. It was reduced by almost 5-fold compared with the PBS group on the 8th day and showed a good antiapoptotic effect. The mRNA transcript expression of *AIF* was also significantly decreased (*P* < 0.01) in the VE and MT groups. In addition, *AIF* expression in the MT group was significantly different (*P* < 0.05) to that in the VE group on the 12th day. Moreover, as an antiapoptosis gene [38], *Bcl-2* mRNA transcript expression levels in the PBS group showed a decline within the first 4 days and then an increase compared with the control group. *Bcl-2* in the VE and MT groups was significantly higher (*P* < 0.05 or *P* < 0.01) than that in the PBS group on the 8th day (Figure 6(g)). Furthermore, the greatest recovery of *Bcl-2* was seen in the MT group on the 8th day compared with the other groups. VE and MT significantly accelerated the upregulation of *Bcl-2* mRNA transcript expression.

## 4. Discussion

Heavy metals contained in food products have a negative impact on human health, as it will be chronic if the food they consume contains heavy metals. The International Codex Alimentarius Commission's (CAC) limit of Cd is 0.05 mg/kg in fruits and vegetables, 0.1 mg/kg in beans and cereals (CAC, CODEX STAN 193-1995). The limit standard of heavy metal residues in food in China stipulates that the limit of Cd concentration is 0.03 mg/kg in fruits, 0.05 mg/kg in vegetables and eggs, and 0.1 mg/kg in aquatic products (limit of contaminants in food, GB2762-2012). However, many aquatic organisms have a very strong enrichment capacity for Cd, and many fish in contaminated areas are seriously overstandard [39]. Even in some places, the Cd content of fish was as high as 37.867 mg/kg [40]. Previous studies found that the LC_50_ of Cd in mice was only 3.2 mg/kg [41] and 15 mg/kg in rabbits [42], which means that residual Cd in some fish may kill the mice or rabbit directly. Therefore, the problem of Cd in fish seriously involves the food safety issues.

Water is the main natural route of exposure to metal pollution in fish. However, our previous study found that large amounts of precipitate covered the surface of the skin and gills and led to dyspnea during Cd water-contaminated exposure. The potential reason for this is not yet clear, but we speculate that Cd may act on the mucus of fish. Therefore, it was difficult to determine whether the death of fish was caused by hypoxia or by Cd poisoning. Moreover, the precipitate also reduced the concentration of Cd in the water environment markedly and may have interfered with the experimental results. To avoid this interference, we delivered the drugs intraperitoneally in the present study.

Currently, to identify therapies, more and more studies have focused on exogenous drugs for Cd-induced damage. In previous studies, VE [43] and MT [44] were used in rats and mice for detoxification. Moreover, VE has also been used in chickens as an antidote [45]. However, to date, these drugs have not been studied in aquatic species with regard to protection against Cd-induced damage. Therefore, we selected exogenous VE and MT to evaluate their protective effects on Cd-induced intoxication in grass carp. The results showed that all the experimental fish demonstrated significant poisoning after the Cd challenge, which resulted in severe oxidative stress and apoptosis in the grass carp liver, with gradual accumulation of Cd in the liver. The accumulation of Cd in the liver reached 51.176 ± 1.070 *μ*g/kg, although accumulation slowed down during the latter part of the Cd challenge. We speculate that a potential reason for this may be due to the body's own detoxification system, such as the antioxidation and self-repair mechanisms. Following Cd challenge, the body's antioxidant enzyme activity increased, and both apoptosis- and antiapoptosis-related gene mRNA transcript expressions changed in order to protect the body against damage. Furthermore, after VE and MT addition, recovery in the detoxification groups was significantly accelerated and similar to the control group within a short period of time. These findings provide a theoretical basis for the treatment and prevention of toxicity caused by heavy metals, which may benefit humans.

Previous studies have shown that Cd is distributed via the blood circulation where it is bound to red blood cells and plasma proteins, mainly albumin, after being absorbed into the body. The liver is a major organ where Cd is distributed [46]. In animal studies, an acute dose of Cd caused severe liver injury and was the major toxic effect [12]. Widespread hepatocellular damage including congestion in mice was caused by Cd [47]. In addition, hepatocellular dissociation, degenerative changes including swelling, hydropic degeneration, hypertrophy and necrosis were seen in the Cd-challenged freshwater fish *Ophiocephalus striatus* [48]. In the present study, severe liver damage including congestion, cytoplasmic dissolution, nuclear debris, and increased inflammatory cell infiltration was observed. This provides further evidence that the liver, which is a sensitive organ, is affected by Cd. Moreover, VE and MT can protect the liver against Cd-induced damage.

Cd can be absorbed into the blood and binds with albumin and other high molecular weight proteins. When the bound Cd is overloaded, free Cd^2+^ will accumulate in various tissues and organs and is eventually delivered to the liver, which is a detoxification organ [49, 50]. However, redundant Cd is likely to cause severe liver damage [51]. In the present study, the Cd content in the liver was measured under different experimental conditions. In our research, the accumulation of Cd in the liver increased with time. However, we also found that Cd then decreased after reaching a certain level in the Cd-challenged grass carp liver. This may be due to the body's self-protection mechanism. The previous study showed that hepatic subcellular Cd was less distributed in nuclei, mitochondria, and microsomes, and more Cd was found in the cytosol in MT-transgenic mice compared with wild-type mice [52]. MT has the ability to chelate [21], therefore, we speculate that the grass carp protects itself by endogenous MT chelating Cd.

Free radicals, which can be generated by Cd, can cause lipid peroxidation [14]. We determined the MDA content to investigate whether Cd caused similar oxidative damage in the grass carp liver. Many studies have shown that MDA concentrations always increase with elevated lipid oxidation, which reflects the level of lipid peroxidation [53] as well as the level of peroxides in the body. Exposure to Cd can result in an excessive generation of MDA [45]. In addition, Cd derived from chicken ovarian follicles showed elevated Cd-induced MDA generation [33]. Moreover, the increase in MDA in broilers caused by Cd at a concentration of 25 mg/L was time-dependent during the 6-week trial [54]. In our research, we also found that MDA was elevated following Cd challenge in grass carp, which indicated that Cd caused severe oxidative damage. VE, as a typical antioxidant, has excellent antioxidative activity. A previous study demonstrated that VE had a protective role in relation to the toxic effects of Cd on hematological indices and lipid peroxide concentration in rats [20]. Furthermore, MT also had an antioxidative effect beyond its chelation function, which reduced the content of free radicals in the rat liver [55]. In the present study, a highly significant reduction in MDA was found in the livers from the VE and MT groups on the 8th day compared with the PBS group, which indicated that these two drugs can recover the antioxidant capacity of the body, and that they can protect the liver by decreasing lipid peroxidation due to the reduced production of MDA. In addition, the content of MDA in the MT group was significantly different to that in the VE group on the 12th day, which indicated that MT had a better effect in reducing the MDA content.

**Figure 7:**
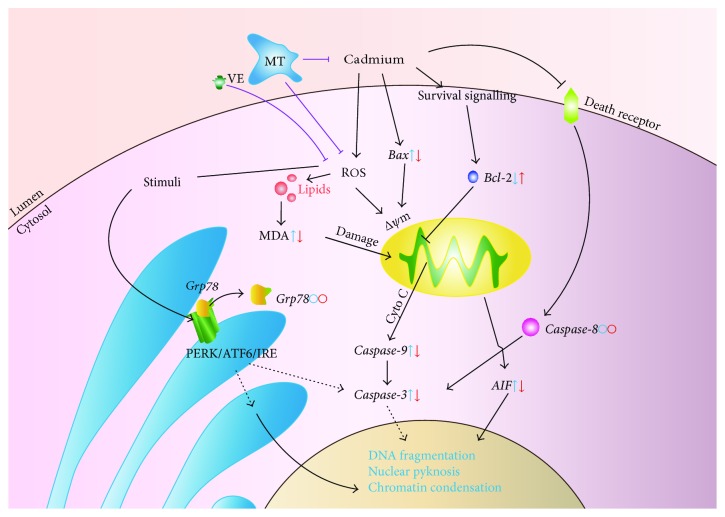
VE and MT protect cells from apoptosis by reducing the Cd content and/or antioxidation. Cd mainly induced apoptosis of liver cells via the induction of the mitochondrial apoptosis pathway rather than the other two pathways; MT mainly inhibited apoptosis by inhibiting Cd accumulation (I) and antioxidation (II), while VE mainly inhibited apoptosis by antioxidation (III). Blue: apoptosis-related gene mRNA transcript expression after Cd challenge, with respect to the controls. Red: apoptosis-related gene mRNA transcript expression after VE/MT supplementation, with respect to the PBS group. The direction of the arrows represents expression of the gene (up or down). The circle represents no significant difference.

We observed cell apoptosis in the Cd-challenged group, which then recovered in the VE/MT groups as shown by histopathology. To further confirm that VE and MT can reduce Cd-induced apoptosis, we determined the apoptotic ratio in each experimental group using flow cytometry. The results showed that VE and MT diminished the apoptosis caused by Cd, and MT was more efficient than VE. Previous research showed that apoptosis is mainly controlled by three major apoptotic pathways: the mitochondrial pathway, death receptor pathway, and endoplasmic reticulum pathway [37]. The apoptotic machinery is well conserved among vertebrates [56]. A similar apoptotic pathway also exists in fish [56–59]. To investigate how VE and MT were regulated to suppress Cd-induced apoptosis, we determined the changes in a number of apoptosis-related genes in the Cd-induced and VE/MT treatment groups. The expression of *caspase-3*, *Bax*, *caspase-9*, *AIF*, and *Bcl-2* was determined to evaluate the apoptotic level associated with the mitochondrial pathway [60–66]. One study suggested that *caspase-8* is located downstream of the death receptor pathway [67]. *Grp78/BiP* is the major endoplasmic reticulum partner with Ca^2+^ binding and antiapoptotic properties in vivo [29]. Our findings showed that Cd induced apoptosis of grass carp liver cells by activating the mitochondrial pathway via upregulation of the expression of *caspase-3*, *Bax*, *caspase-9*, and *AIF* and downregulation of *Bcl-2* expression. We showed Cd-induced apoptosis by inducing the mitochondrial pathway. VE and MT were able to regulate these genes to inhibit apoptosis. Nevertheless, VE and MT had no significant effect on *caspase-8* and *Grp78*/*BiP* mRNA expression, with the exception of a significant effect on the 16th day on *caspase-8* expression. These findings indicated that VE and MT had no obvious effect on the death receptor pathway and endoplasmic reticulum pathway. We conclude that Cd mainly accumulated in cells and initially produced reactive oxygen species then rapidly activated downstream apoptotic genes, which ultimately led to apoptosis. These findings suggested that MT had a stronger hepatoprotective effect than VE in Cd-induced liver injury (Figure 7).

## Figures and Tables

**Table 1:** The LC_50_ of Cd in grass carp.

Concentration of CdCl_2_ (*μ*mol/kg)	Injected concentration (*μ*mol/kg)	The number of deaths	Mortality rate	LC_50_(*μ*mol/kg)
420.709	20	8	80%	199.631
294.994	20	8	80%	
206.845	20	6	60%	
145.036	20	4	40%	
101.696	20	3	30%	
71.3081	20	1	10%	
0	20	0	0	

**Table 2:** Primers of various genes detected with qPCR.

Gene	Abbreviation	Primer sequence (5′–3′)		Acc. number
*B-cell lymphoma 2*	*Bcl-2*	F	GAGATGGCGTCCCAGGTAGAT	JQ713862
		R	GCCAATCCAAGCACTTTCGT	
*Bcl-2 associated X*	*Bax*	F	CAGCCATAAACGTCTTGCGC	JQ793788
		R	GTCGGTTGAAGAGCAGAGTCATTTA	
*Caspase-3*	*CASP3*	F	AGTCGCTGTGCTTCATTTGTTT	JQ793789
		R	CGGTCTCCTCTGAACAGGCTA	
*Caspase-9*	*CASP9*	F	CCTACTCAACCTTTCCAGGCTATG	KT239368
		R	TCATCTGTGGCAACATTCTCCTT	
*Apoptosis-inducing factor*	*AIF*	F	CATGAAGCGAATGATGGAGAAGT	KR872830
		R	CAAAGTCCCTGTAGTTGATGGTGT	
*Glucose-regulated protein-78*	*Grp78/BiP*	F	CTGACCTGAAGAAGTCTGACATCG	FJ436356
		R	GAAGGCTCTTTGCCGTTGAA	
*Caspase-8*	*CASP8*	F	ATGGTAATCTGGTTGAAATCCGTG	KP145003
		R	TCCTTGGCAGGCTTGAATGA	
*β-Actin*	*ACTB*	F	GCTCTGCTATGTGGCTCTTGACT	DQ211096
		R	CAATGGTGATGACCTGTCCGT	
*18S ribosomal RNA*	*RNA18S*	F	ACCCATTGGAGGGCAAGTCT	EU047719
		R	CTCCCGAGATCCAACTACAAGC	
